# A widespread bacteriophage abortive infection system functions through a Type IV toxin–antitoxin mechanism

**DOI:** 10.1093/nar/gkt1419

**Published:** 2014-01-24

**Authors:** Ron L. Dy, Rita Przybilski, Koen Semeijn, George P.C. Salmond, Peter C. Fineran

**Affiliations:** ^1^Department of Microbiology and Immunology, University of Otago, 720 Cumberland Street, PO Box 56, Dunedin 9054, New Zealand and ^2^Department of Biochemistry, University of Cambridge, Tennis Court Road, Cambridge, CB2 1QW, UK

## Abstract

Bacterial abortive infection (Abi) systems are ‘altruistic’ cell death systems that are activated by phage infection and limit viral replication, thereby providing protection to the bacterial population. Here, we have used a novel approach of screening Abi systems as a tool to identify and characterize toxin–antitoxin (TA)-acting Abi systems. We show that AbiE systems are encoded by bicistronic operons and function via a non-interacting (Type IV) bacteriostatic TA mechanism. The *abiE* operon was negatively autoregulated by the antitoxin, AbiEi, a member of a widespread family of putative transcriptional regulators. AbiEi has an N-terminal winged-helix-turn-helix domain that is required for repression of *abiE* transcription, and an uncharacterized bi-functional C-terminal domain, which is necessary for transcriptional repression and sufficient for toxin neutralization. The cognate toxin, AbiEii, is a predicted nucleotidyltransferase (NTase) and member of the DNA polymerase β family. AbiEii specifically bound GTP, and mutations in conserved NTase motifs (I-III) and a newly identified motif (IV), abolished GTP binding and subsequent toxicity. The AbiE systems can provide phage resistance and enable stabilization of mobile genetic elements, such as plasmids. Our study reveals molecular insights into the regulation and function of the widespread bi-functional AbiE Abi-TA systems and the biochemical properties of both toxin and antitoxin proteins.

## INTRODUCTION

The abundance, diversity and importance of bacteriophages (phages) in global biogeochemical and nutrient cycles is undeniable (1,2). Phages are the most numerous biological entities, numbering >10^30^ and participating in 10^25^ infections every second, affecting both bacterial evolution and the turnover of organic matter (2,3). In response, bacteria have developed multiple resistance strategies, including CRISPR-Cas ‘adaptive immunity’ and abortive infection (Abi) ‘innate immunity’ (4–6). Abis are post-infection resistance mechanisms that interfere with phage propagation and result in the death of the infected bacterium—a form of ‘bacterial apoptosis’ (4). The ‘programmed cell death’ that is triggered by Abi systems provides viral protection by limiting phage spread via ‘altruistic cell suicide’ (4,7,8). There are over 20 Abis (predominantly plasmid-encoded lactococcal systems) and, with the exception of a few, the molecular basis for phage resistance is unclear (4).

We recently discovered an Abi (named ToxIN) in *Pectobacterium atrosepticum* that provided high-level resistance against various phages and this bicistronic *toxIN* locus was functional in different genera (9–11). ToxN (toxin) shares sequence homology with AbiQ from *Lactococcus lactis* (12) and is cytotoxic by acting as an endoribonuclease (13,14). Toxicity is inhibited by expression of ToxI (ToxN inhibitor), a repetitive untranslated RNA antitoxin (9,13). ToxIN therefore functions as both an Abi and a toxin-antitoxin (TA) system, and it provided the first direct functional link between Abi and TA systems (9,13). Further, we have demonstrated the same TA properties for related systems from *Bacillus thuringiensis*, *Photorhabdus luminescens*, *Ruminococcus torques*, *Coprococcus catus* and *Eubacterium rectale* (9,11,14). A recent study also showed TA activity for AbiQ from *L. lactis* (15).

TA systems were originally identified on plasmids, where they enhance maintenance by killing cells that lose the plasmid upon division (16). Interest in TAs has increased, due to the realization that they are both widely distributed and abundant in bacterial genomes (16–18). TAs require the dual activity of a toxin and an antagonistic antitoxin. Antitoxins are labile compared with their toxins and, when synthesis of both ceases, the antitoxin is degraded more rapidly, enabling the toxin to affect its target. Currently, five types of TA systems have been defined, based on their mode of antitoxicity (16,17). Type I encode small antisense RNAs that interact directly with the toxin mRNA and inhibit toxin translation (19). In Type II, the protein antitoxin inhibits the toxin by directly binding and forming an inactive TA complex (20). An RNA antitoxin interacts directly with the protein toxin in Type III, of which ToxIN is the defining member (9,13). The CbeA-CbtA Type IV TA encodes an antitoxin that promotes the polymerization of MreB and FtsZ, which are depolymerized by the toxin (21). In the Type V GhoST system, the antitoxin degrades the toxin transcript (22). In all types, the toxin is a protein that targets essential cellular processes, such as DNA replication by inhibiting DNA gyrase (e.g. CcdB) and preventing translation by cleaving mRNAs that are either free (e.g. ToxN) or bound to the ribosomal A-site (e.g. RelE) (16,17).

Diverse roles are proposed for TAs, including management of cellular stress, as selfish genetic elements, as plasmid anti-addiction modules and as mediators of programmed cell death (18). Our recent studies of ToxIN showed that some TA loci are promiscuous phage resistance elements that function upon transfer to new hosts (9,23). There is now mounting evidence that Type I–III TAs, that is *hok*/*sok* (Type I), *mazEF*, *rnlAB* and *lsoAB* (all Type II), can confer virus resistance (9,24–26), suggesting an important evolutionarily role for these loci. Thus far, only ToxIN (Type III) has been shown to function via an Abi (cell suicide) mechanism following phage infection (9).

The discovery of ToxIN set a precedent by showing directly that some Abis function as TAs (9). The Abi-TA link raises the question of whether other Abis provide phage resistance via a TA mechanism. Given the interest in TA systems, bioinformatic studies have assisted the discovery of new Type I-III systems (11,20,27–29). Here, we have taken an experimental approach by examining lactococcal Abis in an attempt to discover new TA loci and to gain greater understanding of Abi mechanisms. We demonstrate that the AbiE phage resistance systems function as novel Type IV TAs and are widespread in bacteria and archaea.

## MATERIALS AND METHODS

### Bacterial strains and culture conditions

Bacterial strains used in this study are listed in Supplementary Table S1. *Escherichia coli* were grown at 37°C in Luria broth (LB) with 200 rpm shaking or on LB containing 1.5% (w/v) agar (LBA). *Streptococcus agalactiae* V/R 2603 was grown at 37°C in Todd-Hewitt broth (THB) without shaking or on THB containing 1.5% (w/v) agar. When relevant, media were supplement with the following antibiotics and additives: ampicillin (Ap) 100 μg/ml; kanamycin (Km) 50 μg/ml; tetracycline (Tc) 10 μg/ml and spectinomycin (Sp) 50 μg/ml. When required, 0.1% (w/v) L-arabinose (ara), 0.2% (w/v) D-glucose (glu) and 1 mM isopropyl-β-D-thiogalactopyranoside (IPTG) were used. Bacterial growth was measured in a Jenway 6300 spectrophotometer at 600 nm (OD_600_). Experiments were repeated in at least three biological replicates.

### DNA isolation and manipulation

All oligonucleotides are outlined in Supplementary Table S2. Plasmid DNA was isolated using the Zyppy Plasmid Miniprep Kit (Zymo). All plasmids are listed in Supplementary Table S3 and were confirmed by DNA sequencing. Restriction digests, ligations, transformation of *E. coli* and agarose gel electrophoresis were performed by standard techniques (30). DNA from PCR and agarose gels was purified using the GE Healthcare Illustra GFX PCR DNA and Gel Band Purification Kit. Restriction enzymes and T4 ligase were from Roche or NEB.

### RNA isolation, RT-PCR and mapping of the *abiE* transcriptional start

Bacteria in exponential phase were pelleted by centrifugation at 2500 ×g for 15 min. Total RNA was isolated using the QIAGEN RNeasy Mini kit. Two micrograms of the extracted RNA was analyzed using a nanodrop ND1000 and on denaturing 1.5% TAE-agarose gels (80 V for 1 h) with 1.5% guanidine-thiocyanite (GTC) to ensure RNA concentration and quality. RNA was DNase treated in solution using the Promega RNAse-free DNase by incubating at 37**°**C for 1 h. RNA was precipitated with 1 volume isopropanol and 0.1 volumes of 3 M NaOAc pH 4.6. The suspension was incubated on ice for 20 min and centrifuged at high speed for 30 min at 4**°**C. The RNA pellet was dried and resuspended with RNase-free MilliQ H_2_O.

For reverse transcription PCR (RT-PCR), 2 μg of total RNA was used for cDNA synthesis using Invitrogen Superscript II First Strand Synthesis kit in a 40 μl reaction. The cDNA was precipitated with 4 volumes of ethanol and 0.2 volumes of 3 M NaOAc pH 4.6. The suspension was centrifuged at 12 000 rpm for 15 min in a benchtop microcentrifuge and the cDNA pellet was dried and resuspended with 10 μl MilliQ H_2_O. To determine the transcription start site, the Roche 5′/3′ RACE kit was used. Briefly, cDNA was polyA-tailed using terminal transferase. Two-steps of PCR amplification using Taq polymerase were performed with nested and adapter primers. The first PCR used Oligo dT and PF1142 primers. The second PCR used the Adapter and PF1143 primers. PCR products were separated on 1% TAE agarose gels, extracted, ligated to pGEM-T-Easy (Invitrogen) and sequenced.

### Toxicity assays

To enable the controlled expression of putative toxins, primers, as listed in Supplementary Table S2, were used to amplify the genes and clone these into pre-digested pBAD30 (Supplementary Table S3). *Escherichia coli* cultures containing pBAD30 derivatives with putative toxins were grown overnight with LB, Ap and glu and sub-cultured to a starting OD_600_ of 0.05 into 25 ml of the same media in 250 ml flasks. Cultures were grown at 37°C with shaking at 200 rpm. When an OD_600_ of ∼0.8 was reached, cells were pelleted and resuspended in LB, Ap and ara to induce toxin expression. At specific time points the OD_600_ was recorded bacteria were pelleted, diluted with phosphate-saline buffer (PBS) and spot-plated on to LBA, Ap and glu for viable counts (colony forming units (cfu/ml)).

### Antitoxicity and bacteriostasis assays

To test for antitoxicity, putative antitoxins were amplified using primers listed in Supplementary Table S2. The products were cloned into pre-digested pTA100 and the resulting constructs introduced into strains containing plasmids for toxin expression. For antitoxicity assays, *E. coli* cultures were grown overnight and sub-cultured into fresh 25 ml LB, Ap, Sp and glu to a starting OD_600_ of 0.05 in 250 ml flasks. After 3 h of growth at 37°C with shaking at 200 rpm, bacteria were pelleted and diluted in PBS and quantitated by plating onto LBA, Ap, Sp plates supplemented with: (i) glu only; (ii) glu and IPTG; (iii) ara only; and (iv) ara and IPTG. For the *L. lactis* AbiE system, bacteria were transferred to LB, Ap, Sp and ara after 3 h of growth and further incubated for 2 h prior to plating for viability. Viable counts were determined after overnight incubation at 37°C and measured as cfu/ml. *Escherichia coli* DH5α, pBAD30, pTA100 was the negative control.

Bacteriostasis was performed using *E. coli* BL21 pRLD25 (*abiEii*-FLAG), pRLD30 (His_6_-*abiEi*) exactly as described for the toxicity assays, with appropriate antibiotics and plating on: (i) LBA, Ap, Sp and glu; and (ii) LBA, Ap, Sp, glu and IPTG. Viable counts were determined after overnight incubation at 37°C and measured as cfu/ml and *E. coli* BL21, pBAD30, pTRB30 was the negative control.

### Plasmid loss assays

The *abiE* operon and promoter were amplified with primers PF1111 and PF1112 and cloned into the EcoRI/HindIII sites of pUC19, resulting in pRLD28. Cultures of *E. coli* DH5α pRLD28 were grown for 24 h in LB without antibiotics. Each day for 5 days, flasks were sub-cultured and dilutions were plated on LBA and incubated at 37°C overnight. To determine plasmid maintenance, 100 colonies were replica plated on LBA, Ap and LBA plates, respectively.

### Co-IP interaction studies

N- and C-terminal hexahistidine-tagged AbiEi were generated with primers PF1138/PF1115 and PF1114/PF1139 and cloned into the BamHI/HindIII or EcoRI/HindIII sites of pTA100, resulting in plasmids pRLD27 (His_6_-AbiEi) and pRLD26 (AbiEi-His_6_). Plasmid pRLD27 (His_6_-AbiEi) was digested with EcoRI/SphI and the His_6_-*abiEi* fragment ligated into EcoRI/SphI-digested pTRB30, giving plasmid pRLD30 (His_6_-AbiEi). Compatible constructs expressing N- or C-terminal FLAG-tagged AbiEii were generated with primers PF1140/PF1112 and PF1113/PF1141. PCR products were cloned into the EcoRI/HindIII sites of pBAD30, giving plasmids pRLD24 (FLAG-AbiEii) and pRLD25 (AbiEii-FLAG).

To examine protein–protein interactions, cultures of *E. coli* BL21, pRLD25, pRLD30 and *E. coli* BL21, pRLD24, pRLD30 were grown overnight with LB, Ap and Km and were sub-cultured into the same medium to a starting OD_600_ of 0.05 in 25 ml in 250 ml flasks. Cultures were grown at 37°C with shaking at 200 rpm. At an OD_600_ of ∼0.8, expression was induced with 1 mM IPTG and 0.1% ara and cultures were incubated for 3 h. Bacteria were pelleted by centrifugation at 3000 ×g at 4°C, resuspended in 1 ml of 0.05 M Tris-HCl, pH 7.4, 0.3 M NaCl (wash buffer) with 10 μl protease inhibitor cocktail (Sigma) and lysed by sonication (6 × 10 s at 30 W with resting on ice between pulses). Fractions were separated by centrifugation at 12 000 rpm for 30 min at 4°C in a microcentrifuge. The soluble fraction was added to 40 μl of pre-washed anti-FLAG agarose and incubated overnight at 4°C with gentle rotation. The anti-FLAG matrix was washed by centrifugation at 8000 rpm for 30 s at 4°C in a microcentrifuge and 1 ml of wash buffer was added. This step was repeated five times and supernatants collected. To elute bound protein(s), 100 μl of 1 × elution peptide (Sigma) was added, incubated on ice for 30 min, centrifuged at 8000 rpm for 30 s at 4°C and the supernatant collected. Fractions were separated on 15% SDS-PAGE gels, blotted and probed for the FLAG and His_6_-tagged proteins as described below.

### Protein purification

*Escherichia coli* BL21, pRLD30 (His_6_-AbiEi) was grown with LB and Km, sub-cultured 1:50 in 500 ml in 2 L flasks and grown at 37°C at 200 rpm. At an OD_600_ of ∼0.5, expression was induced with IPTG and cultures were incubated overnight at 25°C at 200 rpm. Bacteria were pelleted by centrifugation at 3000 ×g at 4°C. Cells were resuspended in 5 ml of 50 mM NaH_2_PO_4_, 300 mM NaCl, 10 mM imidazole, 1 mg/ml lysozyme (Roche), 5 µg/ml DNAse (Roche), and protease inhibitor cocktail (Sigma) and incubated on ice for 30 min. Cells were lysed through three passages in a French press at 16 000/in^2^. The total cell lysate was fractionated by centrifugation at 10 000 ×g at 4°C for 30 min and loaded on Ni-NTA (Qiagen). Unbound proteins were removed by washing with 20 column volumes of 50 mM NaH_2_PO_4_, 300 mM NaCl, 20 mM imidazole and 0.1 µM phenylmethanesulfonylflouride. Proteins bound to Ni-NTA were eluted with 50 mM NaH_2_PO_4_, 300 mM NaCl, 250 mM imidazole supplemented with complete mini-EDTA free (Roche) protease inhibitor cocktail. Proteins were dialysed overnight with 50 mM NaH_2_PO_4_, 300 mM NaCl and stored at 4°C for up to 2 weeks. Fractions were separated on 15% SDS-PAGE gels and coomassie stained or western blots performed to probe for His_6_-tagged proteins as described below.

N- and C-terminal hexahistidine-tagged AbiEii (toxin) were generated with primers PF1198/PF1112 and PF1113/PF1199 and cloned into EcoRI/HindIII-digested pBAD30, resulting in plasmids pRLD55 (His_6_-AbiEi) and pRLD56 (AbiEii-His_6_). Hexahistidine tags did not disrupt toxicity or neutralization by AbiEi (Supplementary Figure S5). AbiEii-His_6_ was purified from cells that co-expressed AbiEi (pRLD69) to enable AbiEii expression without toxicity. *Escherichia coli* BL21, pRLD56, pRLD69 was grown with LB, Km, Ap, glu and sub-cultured 1:50 into 500 ml of LB, Km, Ap IPTG in 2 L flasks. Cultures were grown at 37°C at 200 rpm. At an OD_600_ of ∼0.5, expression was induced with 0.1% ara and incubated overnight at 25°C at 200 rpm. Bacteria were pelleted by centrifugation at 3000 ×g at 4°C. Since AbiEi and AbiEii do not interact, AbiEii-His_6_ was purified separately using Ni-NTA as described above with the purification of His_6_-AbiEi.

### Western blotting

Tagged proteins (His_6_ or FLAG epitope), separated by SDS-PAGE, were transferred to a PVDF membrane (GE Healthcare) using a Criterion blotter (Bio-Rad). The membrane was washed with Western wash buffer (phosphate buffered saline; 0.1% Tween 20) and blocked overnight (with 5% skim milk powder) at 4°C. The membrane was washed 5 × and incubated with the primary antibody for 1 h, washed again (5 ×), incubated with the secondary antibody for 30 min at RT and finally washed again (5 ×). Primary antibodies were mouse monoclonal anti-His (Sigma) or anti-FLAG M2 (Sigma) and the secondary antibody was goat anti-mouse IgG-HRP (Santa Cruz). Proteins were visualized by X-ray film (Kodak) using SignalWest Pico Chemiluminescent substrate kit (Pierce).

### *abiE* promoter activity assays

A truncation plasmid series of the *abiE* promoter fused to a promoterless *lacZ* gene was constructed. First, various promoter lengths were amplified by PCR using the primer pairs PF1111/PF1132, PF1149/PF1132 and PF1150/PF1132, digested with EcoRI/HindIII and cloned into the promoterless *lacZ*-fusion vector pRW50 (31) cut with the same enzymes, creating plasmids pRLD18 (300 bp), pRLD29 (200 bp) and pRLD32 (100 bp), respectively. Next, three pRW50-derived plasmids were generated that had IR1, IR2 or both IR1 and IR2-deleted. The *abiE* promoter was amplified by using primer pairs PF1200/PF1132, PF1150/PF1201 and PF1200/PF1202, which were digested with EcoRI/HindIII and cloned into EcoRI/HindIII-digested pRW50. The resulting plasmids were pRLD75 (ΔIR1), pRLD63 (ΔIR2) and pRLD64 (ΔIR1/2). Finally, reporter plasmids were generated that had IR1, IR2 or both IR1 and IR2 replaced with cytosines. The *abiE* promoter was amplified by using primer pairs PF1202/PF1132, PF1150/PF1203 and PF1202/PF1203, which were digested with EcoRI/HindIII and cloned into EcoRI/HindIII-digested pRW50. The resulting plasmids were pRLD65 (IR1C), pRLD67 (IR2C) and pRLD68 (IR1/2C). β-galactosidase promoter activity assays were performed in *E. coli* DH5α as described previously (32). β-galactosidase activity was expressed in Miller Units (MU) (33).

### DIG-labelled EMSAs

The DIG gel shift kit (2nd Generation, Roche) was used as described by the manufacturer. The promoter region of *abiE* was amplified by PCR using the same primers used for making the *lacZ*-reporter plasmids described earlier. Products were purified and 3′ end labelled with DIG-11-ddUTP using terminal transferase. The reaction was diluted 1:10 to a final concentration of 0.4 ng/μl DIG-labelled DNA. As a non-specific control, a CRISPR-repeat-spacer unit from *Serratia* sp. ATCC 39006 was used (primers PF926/PF922). Electrophoretic mobility shift assay (EMSA) reactions (10 μl) contained 2 μl binding buffer (100 mM Hepes, pH 7.6, 5 mM EDTA, 50 mM (NH_4_)_2_SO_4_, 5 mM DTT, 1% w/v Tween 20, 150 mM KCl), 0.5 μl Poly d[I-C], 1 μl DIG-labelled DNA (0.4 ng/μl), varying amounts of protein and unlabelled DNA. Reactions were incubated at room temperature for 15 min, separated on pre-run (5 min) 6% TAE polyacrylamide gels and transferred onto Amersham Hybond-N nucleic acid membranes (GE Healthcare) using a Criterion blotter (Bio-Rad). Membranes were fixed at 120°C for 30 min and detection performed and visualized by X-ray film (Kodak).

### Construction of SDM of AbiEii

To construct alanine or glutamate site-directed mutants (SDM) of various conserved amino acids in AbiEii, an overlap extension PCR strategy was used (34). The left flanks were amplified by PCR using PF1113 as the forward primer and the specific reverse mutant primers (Supplementary Table S2). For the G50A and G49A/G50A mutants the G49A reverse primer was used (PF1178), for the D69A, D67A/D69A, D67E and D69E mutants the D67A reverse primer was used (PF1184) and for the D192E mutant the D192A reverse primer was used (PF1194). The right hand flanks were amplified with the specific forward mutant primers, which included the required codon change (Supplementary Table S2), and the reverse primer PF1112. Overlap extension PCR was performed using 1 μl of each fragment as template and primer combinations PF1113/PF1112 and PF1113/PF1141 for the native and C-terminally FLAG-tagged version of these site-directed AbiEii mutants, respectively. The overlapped products were digested with EcoRI and HindIII and cloned into EcoRI/HindIII-digested pBAD30. All SDM plasmids are listed in Supplementary Table S3. To generate SDM plasmids for protein purification, C-terminal His_6_-tags were added. Primers PF1113 and PF1199 were used to amplify *abiEii* mutants from plasmids pRLD38, pRLD49 and pRLD43, which were cloned into EcoRI/HindIII-digested pBAD30, giving plasmids pRLD70 (G49A, G50A AbiEii-His_6_), pRLD72 (D67A, D69A AbiEii-His_6_) and pRLD73 (D192A AbiEii-His_6_).

### *In vitro* radioactive GTP binding assay

Purified AbiEii-His_6_ was incubated with 0.65 pmol of [α-^32^P]-NTP in binding buffer (10 mM Tris-HCl, 1.5 mM MgCl_2_, 50 mM KCl, pH 8.3) at 37°C for 1 h. Radioactive ATP, GTP, CTP and UTP ([α-^32^P]-NTP; Perkin and Elmer) had the following properties: [α-^32^P], 3000 Ci/mmol 10 mCi/ml and 250 μCi. For competition assays, 65 μmol of unlabelled nucleotides were added to the binding reaction before the addition of the AbiEii. Samples were run on a 6% TAE native polyacrylamide gel for 3 h at 100 V. Gels were exposed overnight to a phosphoimager cassette and radioactivity detected using a Personal Molecular Imager System (Bio-Rad).

## RESULTS

### AbiE constitutes a TA system

**Figure 1.**
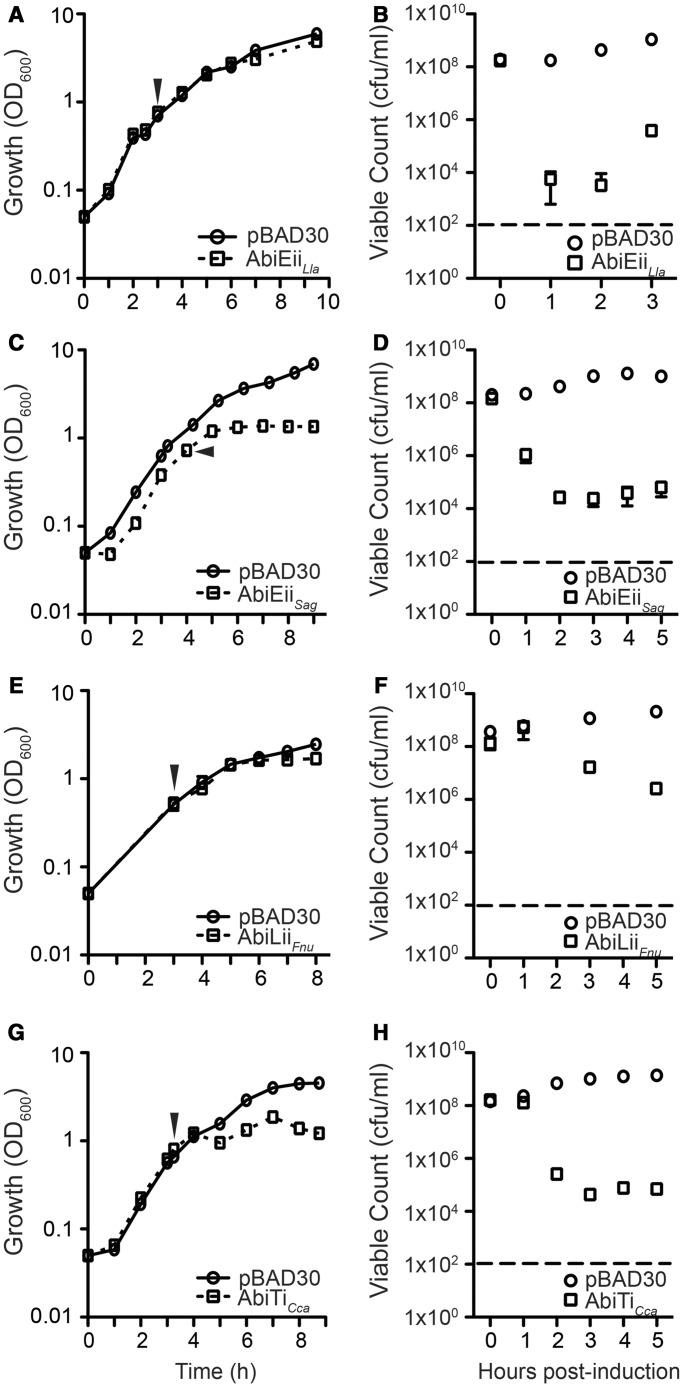
*Lactococcus lactis* AbiEii, *S. agalactiae* AbiEii, *F. nucleatum* AbiLii and *C. catus* AbiTi are toxic. Growth and viability of *E. coli* following induction (arrow) of *L. lactis abiEii* (pRLD20) (**A**, **B**), *S. agalactiae abiEii* (pRLD12) (**C**, **D**), *F. nucleatum abiLii* (pRLD15) (**E**, **F**) and *C. catus abiTi* (pRLD22) (**G**, **H**). Plasmid pBAD30 was the negative control in all experiments and the dashed lines indicate limit of detection. Data shown are means ± SD of three biological replicates.

In common with many TAs, some Abis are organized in bicistronic operons. AbiE, AbiG, AbiL and AbiT are encoded by *abiEi*/*abiEii*, *abiGi*/*abiGii*, *abiLi*/*abiLii* and *abiTi*/*abiTii*, respectively, and are putatively transcribed as bicistronic operons (35–39). In addition, AbiU consists of *abiU1* and *abiU2* (40) and some Abis exhibit toxicity (i.e. AbiB (4), AbiD1 (41,42), AbiK (43), AbiN (44), AbiO (45), AbiQ (9,12) and AbiRa-c (46)). Theoretically, these Abis might have RNA antitoxins (i.e. be Type I or III TAs), such as the case of AbiQ/ToxIN (9,15). To test if some Abis are also TAs, we individually cloned all lactococcal two-component Abis; *abiEi*, *abiEii*, *abiGi*, *abiGii*, *abiLi*, *abiLii*, *abiTi*, *abiTii* and *abiU2*, into tightly controllable expression vectors and screened for toxicity in *E. coli*. Only AbiEii from *L. lactis* inhibited growth (Figure 1A and B, and Supplementary Figure S1). In addition, we tested the toxicity of homologues of AbiE, AbiT and AbiL from other bacteria. AbiEii from *Streptococcus agalactiae* (Sag) V/R 2603, AbiLii from *Fusobacterium nucleatum* (Fnu) and AbiTi from *C. catus* (Cca) GD7 also inhibited growth (Figure 1C–H). To examine if other single gene Abis were toxic, AbiB, AbiF, AbiJ and AbiO were expressed in *E. coli*, but none detectably inhibited growth (Supplementary Figure S2).

**Figure 2.**
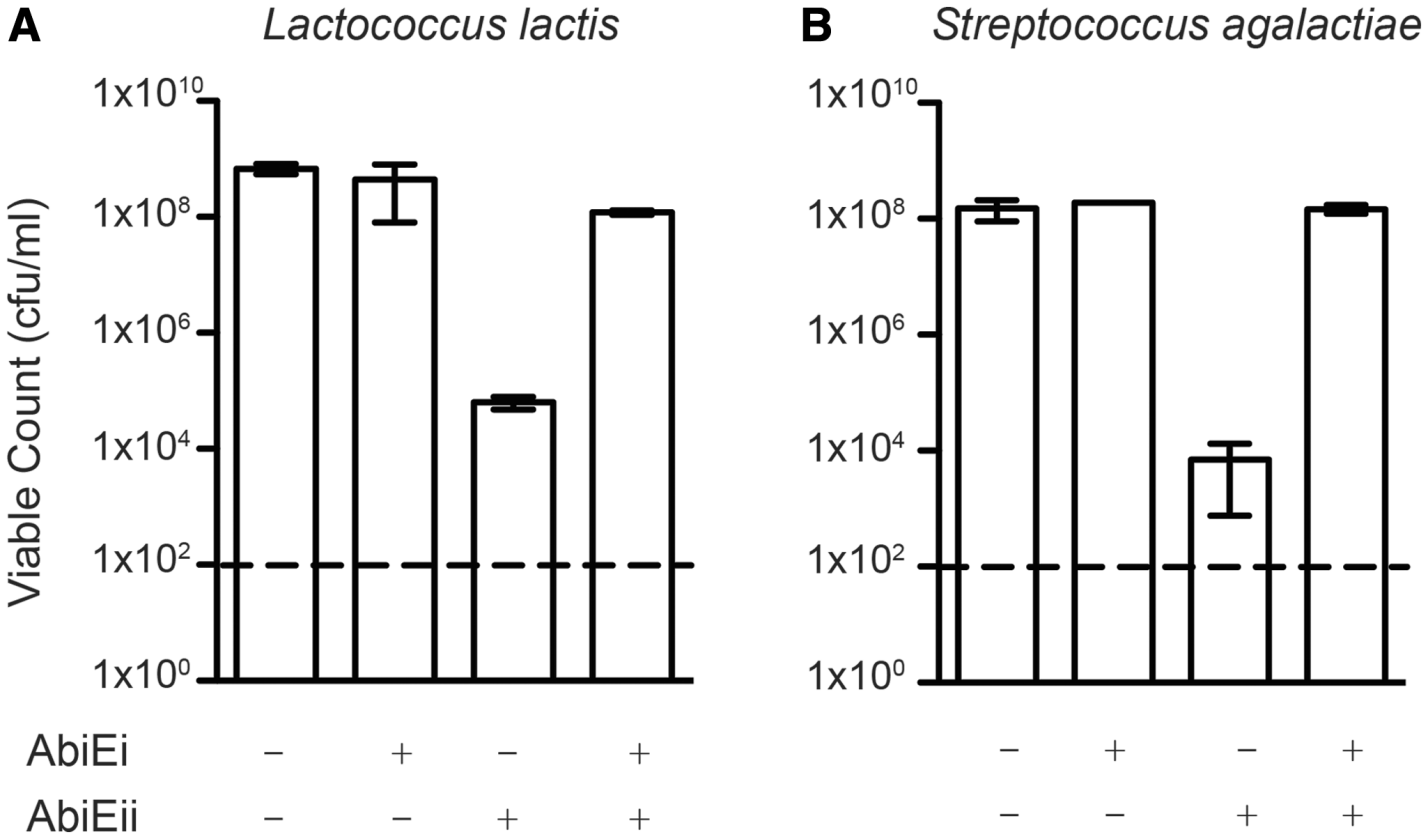
*Lactococcus lactis* and *S. agalactiae* AbiEi and AbiEii are TA systems. (**A**) AbiEi from *L. lactis* protects from AbiEii-mediated toxicity. Antitoxicity assays of *E. coli*, pBAD30, pTA100 (control); *E. coli*, pBAD30, pRLD21 (*L. lactis* AbiEi/antitoxin); *E. coli*, pRLD20 pTA100 (*L. lactis* AbiEii/toxin) and *E. coli*, pRLD20 pRLD21 (*L. lactis* AbiEi & AbiEii/toxin and antitoxin). (**B**) AbiEi from *S. agalactiae* protects from AbiEii-mediated toxicity. Antitoxicity assays of *E. coli*, pRLD12, pRLD13 (*S. agalactiae* AbiEi and AbiEii). In (B) symbols + and – refer to induction or repression of *abiEii* (ara and glu) and *abiEi* (+/– IPTG). Dashed lines indicate the limit of detection. Data shown are means ± SD of three biological replicates.

To test adjacent genes for antitoxicity, expression of putative antitoxins from a second plasmid was assessed. For AbiLi*_Fnu_* and AbiTii*_Cca_*, no antitoxicity was observed against AbiLii*_Fnu_* and AbiTi*_Cca_*, respectively (Supplementary Figure S3). In contrast, expression of AbiEi from *L. lactis* or *S. agalactiae* counteracted the growth inhibitory phenotypes of their respective AbiEii proteins (Figure 2). In summary, in addition to AbiQ/ToxIN, at least one additional Abi family, namely AbiE, may act as a TA module.

### AbiE is widespread in bacterial genomes and extrachromosomal elements

AbiE of *L. lactis* plasmid pNP40 was the first two-component Abi identified and it aborts the 936 phage family, preventing DNA packaging through an unknown mechanism (36,47,48). AbiE in the human pathogen *S. agalactiae* consists of SAG1284 (*abiEi*) and SAG1285 (*abiEii*), but these are misannotated as *abiG*, to which we could find no similarity. This annotation is prevalent through the sequence databases, with many *abiE* homologues denoted as *abiG*. AbiEi and AbiEii of *L. lactis* and *S. agalactiae* share ∼25% amino acid identity (Supplementary Figure S4). The *S. agalactiae abiE* is within an integrative and conjugative element (ICE), termed ICE*Sa2603rplL*, which encodes virulence and metal resistance genes (49). This ICE is conserved among β-hemolytic streptococcal groups B, C and G and is transmissible to group A and α-hemolytic streptococci (50,51). Versions of this island differ in size, ranging from ∼50–90 kb (Supplementary Table S4) and typically encode antibiotic resistance genes (50). AbiE encoded by these elements might assist maintenance in addition to phage resistance (9,14).

**Figure 3.**
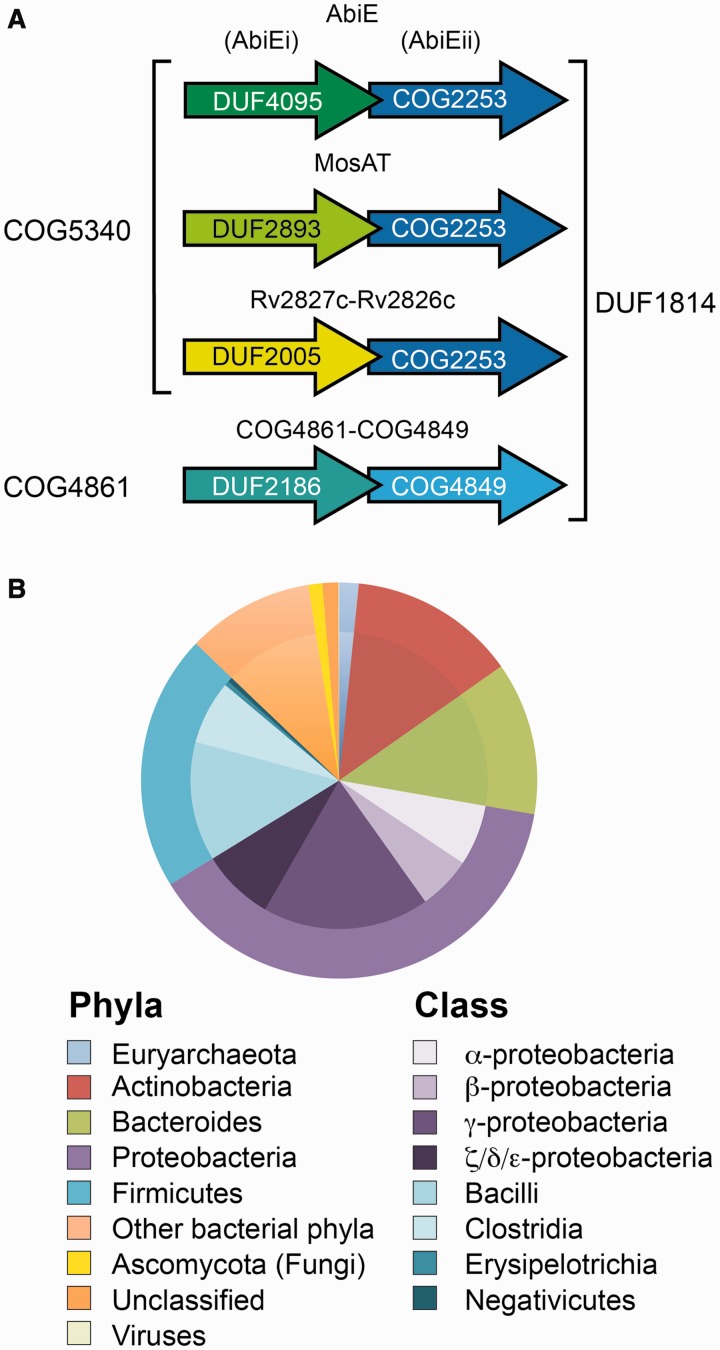
AbiE is a widely distributed bicistronic operon. (**A**) Gene arrangements of the several AbiEi antitoxin families and AbiEii homologues. (**B**) The taxonomic distribution profile of the DUF1814 protein superfamily is shown as a piechart, with the outer ring representing the Phyla and the inner ring representing the Class.

AbiEi and AbiEii are members of abundant uncharacterized protein families that form two highly associated protein pairs. Our analysis described below, led to the prediction that AbiEi was a transcriptional regulator and AbiEii a nucleotidyltransferase (NTase). AbiEi belongs to the cluster of orthologous group COG5340 (domain of unknown function DUF4095) and AbiEii is a DUF1814 protein. The DUF1814 family consists of COG4849 and COG2253 proteins. Specifically, AbiEii is a COG2253 member. Genes encoding other DUFs reside upstream of COG2253 genes, such as DUF2893 and DUF2005 (Figure 3A), which are COG5340 members. DUF1814 genes are usually located adjacent to COG5340 or COG4861 genes (Figure 3A). Specifically, COG5340 is associated with COG2253 and COG4861 is linked to COG4849 with STRING protein-association scores of 82% and 99%, respectively (52). Although COG5340 and COG4861 do not share sequence identity, both are putative transcriptional regulators. DUF1814 proteins are widespread, present in ∼3000 bacterial, archaeal and even fungal genomes (Figure 3B). Not all genes encoding DUF1814 proteins are genetically linked to putative antitoxins, which is the case for the fungal homologues. COG2253, COG4849 and DUF1814 proteins have been proposed as novel families of uncharacterized NTases (53,54). In summary, analysis of AbiE led to the identification of widespread and highly associated gene pairs COG5340-COG2253 and COG4861-COG4849. In each case, a gene encoding a predicted transcriptional regulator precedes a gene for a putative NTase.

### AbiE provides plasmid stabilization

**Figure 4.**
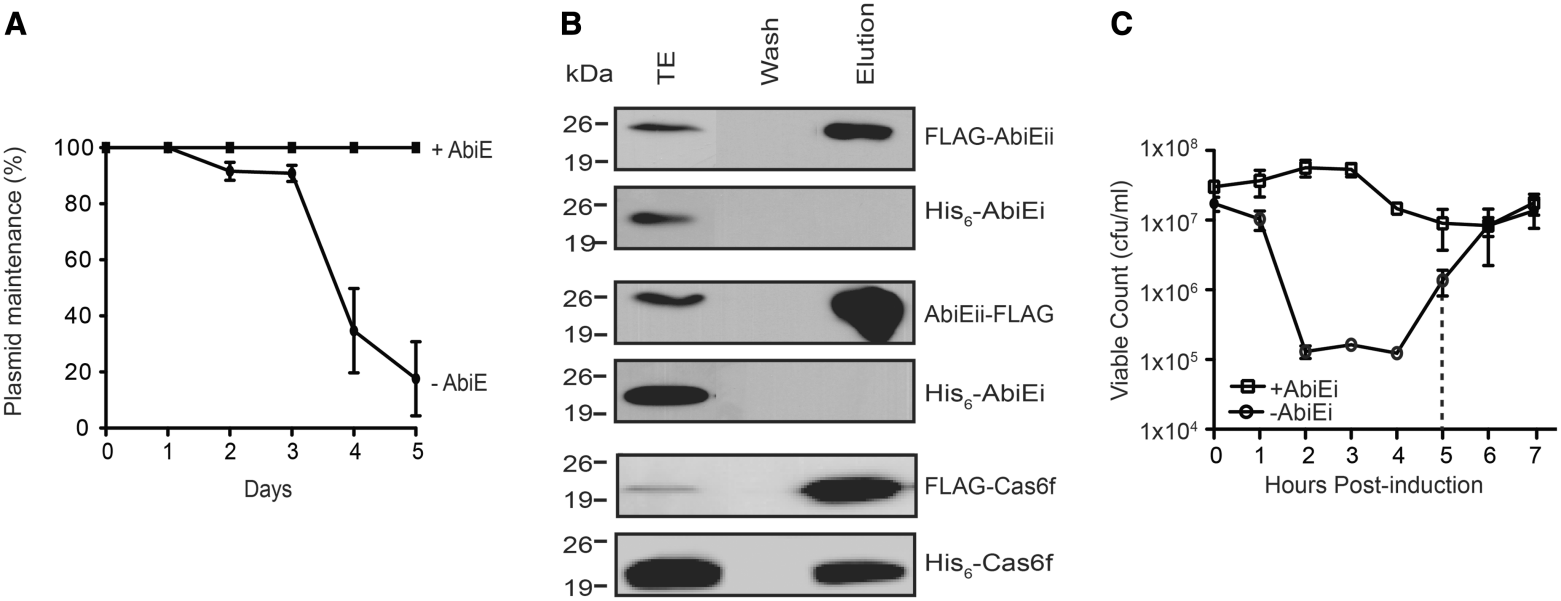
AbiE is a bacteriostatic type IV TA system that stabilizes plasmids. (**A**) *Escherichia coli* carrying pUC19 with and without the *abiE* operon (pRLD28 and pUC19, respectively) was assayed for plasmid maintenance without antibiotic selection. Data shown are means ± SD of three biological replicates. (**B**) AbiEi and AbiEii do not interact. Western blot of the total cell extract (TE), final wash (Wash) and elution (Elution) fractions of Co-IP assays. Two assays were performed with N- or C- FLAG-tagged AbiEii as bait (pRLD24 and pRLD25, respectively) and His_6_-AbiEi (pRLD30) as prey in *E. coli*. A positive control included FLAG-Cas6f (pJSC6) and His_6_-Cas6f (pTG116) proteins that interact robustly (50). (**C**) Bacteriostasis assays with *E. coli* BL21, pRLD25 (AbiEii-FLAG), pRLD30 (His_6_-AbiEi) were performed. After induction of AbiEii expression, viable counts were determined on LBA Ap, Sp without (AbiEii), or with (AbiEi+AbiEii), IPTG. Dashed lines indicate the limit of detection. Data shown are means ± SD of three biological replicates.

We decided to investigate the *S. agalactiae* AbiE further due to the strong TA phenotype and the tight control we could achieve using this system. One role of TAs is to maintain extrachromosomal elements, such as plasmids. The *abiE* operon provides phage resistance (36) and is present in mobile genetic elements and plasmids. Thus, like *toxIN*, *abiE* may have roles in addition to providing phage protection. Plasmid pUC19 exhibited ∼80% loss from *E. coli* when grown without selection for 5 days, whereas, no plasmid loss occurred when the *abiE* operon and native promoter were introduced into pUC19 (Figure 4A). Therefore, the AbiE system enables plasmid maintenance.

### AbiE is a Type IV TA system

We considered three possible mechanisms to account for AbiEi antitoxicity: (i) formation of an inactive complex with AbiEii (Type II); (ii) acting on the same target as AbiEii (Type IV); or (iii) functioning as an endoribonuclease to degrade *abiEii* transcripts (Type V). To investigate if AbiE was a Type II system, we tested if AbiEi and AbiEii interact. His_6_-tags were added to N- or C-terminal ends of AbiEi and FLAG tags were added to N- or C-terminal ends of AbiEii. Protein function was assessed in kill/rescue assays, which showed that C-terminal FLAG-tagged AbiEii (AbiEii-FLAG) was toxic and both N- and C-terminally His_6_-tagged AbiEi constructs were antitoxic (Supplementary Figure S5A). FLAG-AbiEii was non-toxic, despite being detected by western blot (Supplementary Figure S5B and D). In contrast, AbiEi-His_6_ was not detected, so was omitted even though it was functional (Supplementary Figure S5A and D). Since it was not known where AbiEi binds to AbiEii, if at all, both FLAG-AbiEii and AbiEii-FLAG were included in co-immunoprecipitations (Co-IP). Co-IP was performed with the FLAG-tagged AbiEii proteins as bait and His_6_-tagged AbiEi proteins as prey and no interactions were detected (Figure 4B). We performed a technical positive control using Cas6f (previously Csy4) from *P. atrosepticum* (Figure 4B), which demonstrated the vector set-up and Co-IP was functional (32). Therefore, AbiE is not a Type II system. Furthermore, since AbiEii is detected upon co-overexpression of the antitoxin AbiEi (Figure 4B), which contains no predicted ribonuclease activity, AbiE cannot be a Type V system. We conclude that AbiE is a new member of the recently described non-interacting Type IV TA systems.

### AbiE is a reversible bacteriostatic TA system

Toxins elicit either a reversible growth arrest or cell death. To understand the mechanism of AbiEii/DUF1814 proteins, we investigated if toxicity was bactericidal or bacteriostatic. *Escherichia coli* containing separate AbiEi and AbiEii expression plasmids were grown and the toxin induced. The colony forming ability was assessed hourly on media that either repressed toxin production (LBA and glu) or enabled the delayed ‘rescue’ due to antitoxin expression (LBA, glu and IPTG). It took between 1 and 2 h for AbiEii to cause the maximal inhibitory effect on growth (Figure 4C). For up to 4 h post-AbiEii induction, delayed expression of AbiEi rescued viability to levels similar to the initial cell numbers (Figure 4C). However, after 4 h, spontaneous AbiEii-defective plasmid mutants arose (Figure 4C, dashed line). No changes in morphology or viability were detected using LIVE/DEAD staining and TEM (Supplementary Figure S6). Therefore, AbiEii does not affect morphology and is reversibly bacteriostatic by direct (Figure 2B) or latent expression of its cognate antitoxin.

### *abiEi* and *abiEii* are bicistronic

**Figure 5.**
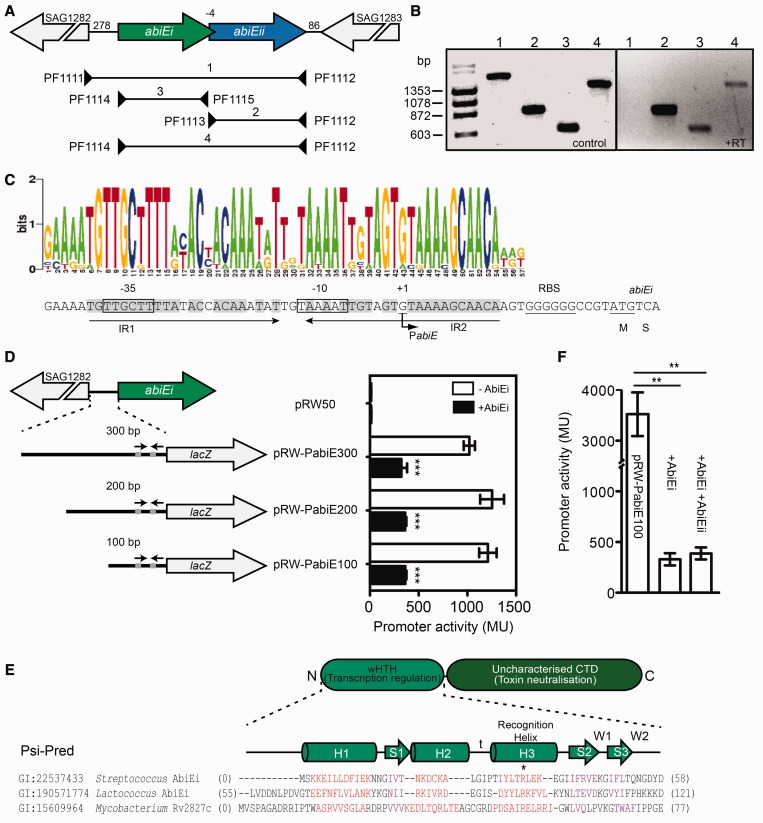
AbiEi contains a wHTH domain and is negatively autoregulatory. (**A**) Schematic diagram of the genetic organization of *abiE* in the *S. agalactiae* V/R 2603 genome (not to scale). Triangles depict the RT-PCR primers. (**B**) *abiEi* and *abiEii* are operonic. The PCR control used *S. agalactiae* as the template. +RT indicates PCR products from cDNA reversely transcribed from *E. coli* DH5α, pRLD16 total RNA. (**C**) The transcriptional start site of *abiE* was mapped (+1 and arrow). The putative –10 (TAAAAT) and –35 (TTGCTT) are boxed and the ribosome binding site (RBS) and the translational start site of AbiEi are underlined. The palindromic repeats are denoted IR1 and IR2 (arrows and grey shading). The Weblogo consensus of the inverted repeats and promoter region is aligned above (see Supplementary Figure S8). (**D**) Schematic diagram of the promoter truncations made to assess P*_abiE_* activity (left)*.* Activity of the three *abiE* promoter truncations, 300 bp (pRLD18), 200 bp (pRLD29) and 100 bp (pRLD32), in the presence (pRLD13) or absence (pTA100) of AbiEi (right). (**E**) Schematic diagram of the domain organization of AbiEi, and protein sequence alignment and secondary structure of the N-terminal domain of AbiEi with its homologue Rv2827c showing the wHTH domain. (**F**) Promoter activity of the 100 bp truncation (pRLD32) was assessed with repression by the AbiEi antitoxin (pRLD13) in the absence (pBAD30) or presence of AbiEii toxin expression (pRLD12). *P-value <0.05, **P-value < 0.01 and ***P-value < 0.005 (two-tailed *t*-test). Data shown are means ± SD of three biological replicates.

*Streptococcus agalactiae abiEi* overlaps the 5′ end of *abiEii* by 4 nt and RT-PCR revealed transcripts that covered both genes, demonstrating a bicistronic arrangement for *abiEi* and *abiEii* (Figure 5A and B). To understand the regulation, the transcriptional start (+1) of *abiE* was mapped and was located 26 bp 5′ of the *abiEi* ATG start codon. Putative –10 and –35 elements were proposed, separated by 17 bp (Figure 5C). Intergenic truncation experiments demonstrated that this region contained an active promoter (Figure 5D, white bars). Therefore, AbiE is encoded by a bicistronic operon transcribed from a promoter within ∼60 bp of the translational start site.

### AbiEi negatively autoregulates the *abiE* promoter

Negative autoregulation is a common feature of Type II TAs, but is not yet established for Type IV systems. Autoregulation is mediated by the antitoxin alone, or in concert with the toxin, and serves to control TA levels. AbiEi (COG5340/DUF4095) and homologues (DUF2893) were similar to Rv2827c from *Mycobacterium tuberculosis* (∼90% coverage) (55). Rv2827c is a COG5340/DUF2005 member and the adjacent gene, *rv2826c*, encodes a DUF1814 protein. Rv2827c and AbiEi-related proteins possess two domains: an N-terminal winged-helix-turn-helix (wHTH) (56) and an uncharacterized C-terminal domain (CTD) (Figure 5E). The wHTH has a secondary structure of H1-S1-H2-t-H3-S2-W1-S3-W2 (H = helix, S = strand, and W = Wing) (Figure 5E). COG4849, another putative antitoxin (Figure 3A), also contains a wHTH. H3 is the positively charged recognition helix and embeds into the major groove of DNA for sequence binding and recognition (Figure 5E) (56). AbiEi autoregulates, since AbiEi repressed transcription of P*_abiE_*-*lacZ* fusions (Figure 5D, black bars). To determine if AbiEi and AbiEii act synergistically, both were overexpressed, but no additional repression was detected (Figure 5F). In summary, AbiEi regulates the *abiE* promoter, and this is the first indication that Type IV antitoxins negatively autoregulate.

### Full-length AbiEi is required for autoregulation and the CTD is sufficient for antitoxicity

**Figure 6.**
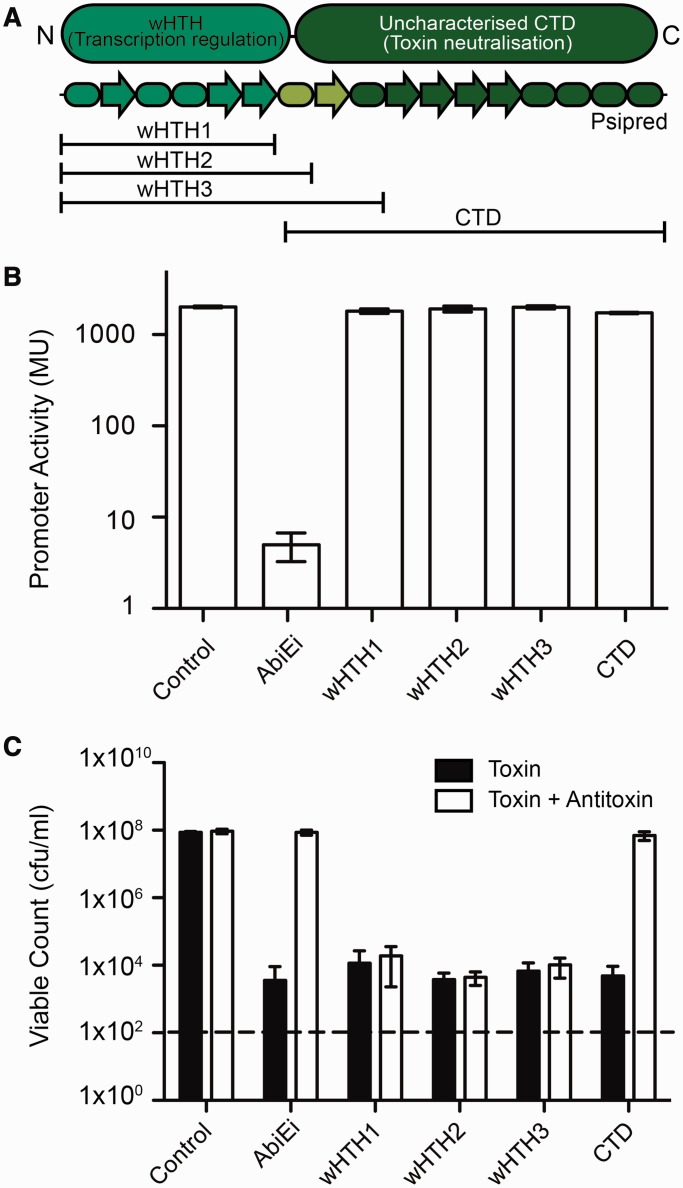
Full-length AbiEi is required for autoregulation and the CTD is sufficient for antitoxicity. (**A**) Schematic diagram of the domain organization and secondary structure of AbiEi, and the truncations made to assess autoregulation and antitoxic functionalities. (**B**) Promoter activity of the 100 bp P*_abiE_* was assessed in the absence (pTA100) or presence of AbiEi (pRLD13) or the four AbiEi truncations. (**C**) The CTD of AbiEi provides complete antitoxicity against AbiEii. Antitoxicity assays of AbiEii (pRLD12) with AbiEi or the four AbiEi truncations. Black bars represent viable counts from plating on to LBA, Ap, Sp and ara. White bars represent viable counts from plating on to LBA, Ap, Sp, ara and IPTG. Viable counts from plating on to LBA, Ap, Sp, glu are shown in Supplementary Figure S7. Refer to Supplementary Table S3 for plasmid details. Data shown are means ± SD of three biological replicates.

Given that AbiEi contains both a wHTH and an uncharacterized CTD, we investigated the roles of each domain in regulation and antitoxicity. Using a structural alignment of AbiEi against Rv2827c, a series of native and His_6_-tagged truncation mutants were generated, which contained either the wHTH or the CTD (Figure 6A) and these were tested for stability by western blotting. None of the truncation mutants were autoregulatory, but they were unstable, indicating that the full-length protein is necessary for repression and/or stability (Figure 6B, Supplementary Figure S7A and B). However, the CTD alone was sufficient for inhibition of toxicity, whereas the NTD truncations were non-protective (Figure 6C, Supplementary Figure S7C and D). Therefore, the full-length protein is likely to be required for *abiE* repression, and the CTD is bi-functional, contributing to repression and with an independent role in antitoxicity.

### AbiEi binds inverted repeats and represses the *abiE* promoter

**Figure 7.**
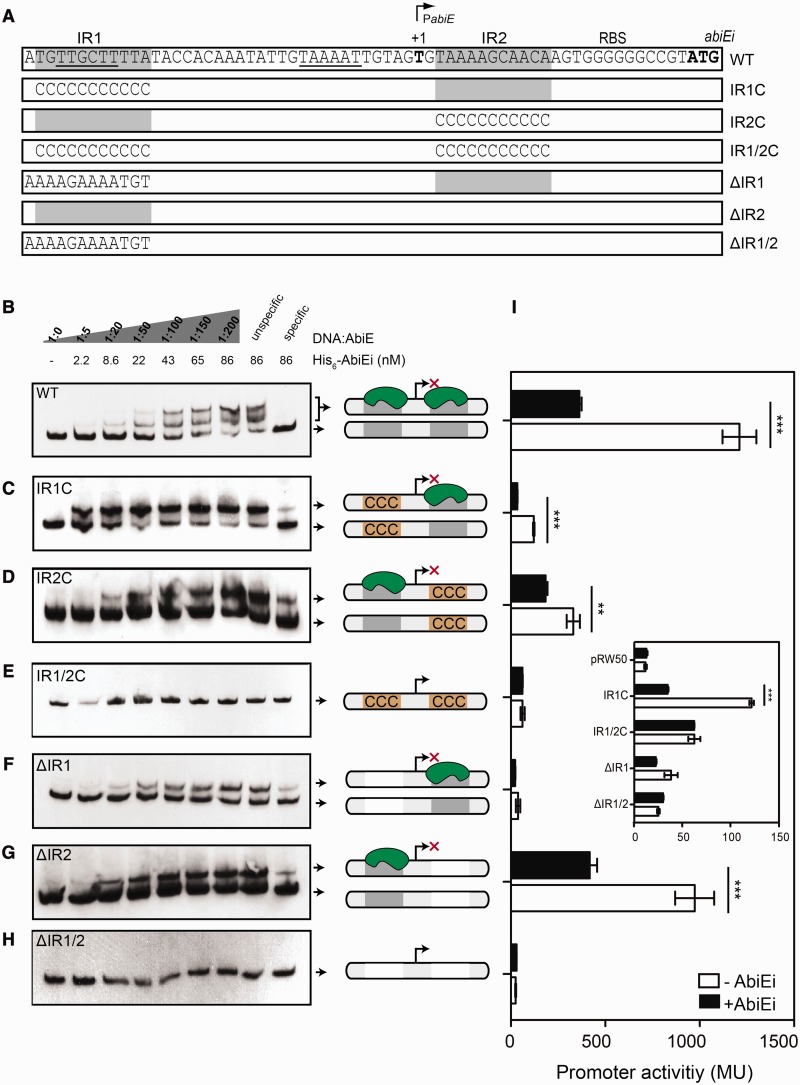
AbiEi negatively autoregulates by binding specifically to two IRs. (**A**) Diagram of the promoter-mutant constructs detailing the mutations made. The promoter features include the –10 and –35 (underlined), the two IR elements (grey boxes), RBS and +1. (**B–H**) EMSAs of increasing concentrations of purified His_6_-AbiEi with the 100 bp DIG-labelled *abiE* promoter DNA and specific IR replacements (IR1C, IR2C and IR1/2C) and deletions (ΔIR1, ΔIR2 and ΔIR1/2). (**I**) Promoter activities of the 100 bp P*_abiE_* truncation and mutant derivatives were assessed in the presence (pRLD13) or absence (pTA100) of AbiEi. Refer to Supplementary Table S3 for the plasmid details. *P-value < 0.05, **P-value < 0.01 and ***P-value < 0.005 (unpaired two-tailed *t*-test). Data shown are means ± SD of three biological replicates.

Winged-HTH proteins bind to repetitive DNA elements (56). The *abiE* promoter contains a perfect 11 bp palindromic repeat of 5′-TGTTGCTTTTA-N_27_-TAAAAGCAACA-3′, which we termed inverted repeat 1 (IR1) and IR2 for each site (Figures 5C and 7A). Furthermore, these IR extend up to 23 bp with only 4 mismatches (5′-TGTTGCTTTTATACCACAAATAT-N_3_-AAAATTGTAGTGTAAAAGCAACA-3′) and similar IRs were identified upstream of other *abiE* loci (Supplementary Figure S8). To examine if AbiEi bound directly to the *abiE* promoter, EMSAs were performed. His_6_-AbiEi and AbiEi-His_6_ were antitoxic and repressed the P*_abiE_*-*lacZ* fusion, demonstrating no loss of function after tagging (Supplementary Figures S5A and S7A). AbiEi-His_6_ was not detectable by western blot (Supplementary Figure S5D), so His_6_-AbiEi was used in EMSAs. Two protein:DNA complexes were detected with increasing His_6_-AbiEi concentrations, indicative of two potential DNA-binding sites (Figure 7A and B). The binding of AbiEi to DNA upstream of *abiE* was specific since excess specific, unlabelled DNA reversed the shift, whereas an excess of non-specific DNA had no effect. Toxin addition did not affect the banding profile (data not shown). Consistent with the *lacZ*-reporter data, the toxin had no detectable role for regulation and AbiEi bound directly to the *abiE* promoter to cause repression.

To determine the role of the IRs, we assessed if AbiEi could bind to promoter variants with the IRs either deleted or replaced with cytosines. Deletion or replacement of IR1 or IR2 resulted in a single shift (Figure 7C, D, F and G), demonstrating disruption of the second binding site. Binding affinities (K_d_) for the WT and IR-mutants ranged from 10 to 30 nM, suggesting non-cooperative binding (Supplementary Figure S9). The deletion or replacement of both IR sites completely abolished AbiEi binding (Figure 7E and H).

To test if IR1 and/or IR2 were important for AbiEi-directed repression, IR1 and IR2 were deleted or replaced in P*_abiE_*-*lacZ* fusion plasmids. Deletion of IR1 severely attenuated activity, due to removal of the –35 element (Figure 7I). When IR1 was replaced with cytosines (IR1C), overall promoter activity was reduced, due to changes in the –35 sequence (Figure 7A). However, AbiEi repressed the IR1C variant, due to binding at IR2 (Figure 7I). Expression of the IR2-deleted or cytosine-replaced promoter variants was decreased relative to the WT, but they were still repressed by AbiEi. Both IR2 mutant promoter reporters resulted in higher %GC content after the +1 (Figure 7A), which might decrease open complex formation during transcription initiation. Deletions or substitutions in both IR1 and IR2 resulted in negligible expression, which could not be repressed (Figure 7I, inset). Together, our data show that AbiEi is a transcriptional autorepressor that independently binds directly to two conserved IR sequences in the *abiE* promoter.

### AbiEii is a member of the DNA polβ NTase superfamily

**Figure 8.**
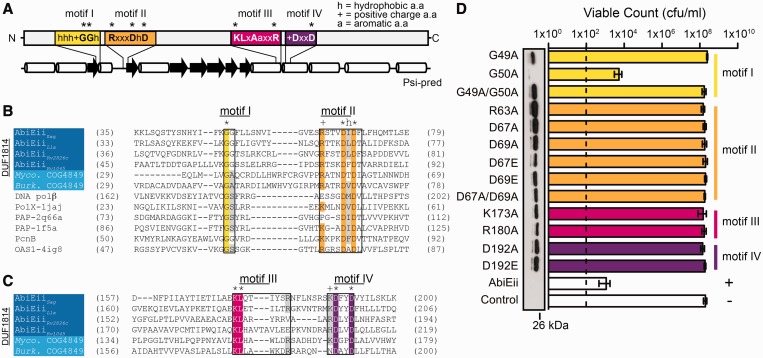
AbiEii is a toxin member of the DNA polβ superfamily. (**A**) Secondary structure prediction of AbiEii highlighting the four conserved motifs. The cylinders depict α helices and arrows depict β strands. Residues indicated by asterisks denote residues selected for SDM. Protein sequence alignments of (**B**) motifs I and II regions of AbiEii and homologues from COG2253 and COG4849 families and (**C**) motifs III and IV exclusive to the AbiEii-DUF1814 protein family. *Myco* depicts sequences from *M. tuberculosis*, and *Burk* from *Burholderia multivorans*. DNA polβ proteins include the human DNA polβ polymerase, Polymerase X from swine virus (PolX, PDB: 1jaj), PAP from yeast (PDB: 2q66a), mammalian (PDB: 1f5a) and bacterial (PcnB) cells, and the human oligoadenylate synthetase (OAS1, PDB: 4igb). (**D**) Mutation of the conserved motifs (I–IV) in AbiEii abolished toxicity. Toxicity (cfu/ml) was determined for *E. coli* with plasmids expressing the AbiEii variants. The dashed line indicates limit of detection. Data shown are means ± SD of three biological replicates. Western blots were performed on culture samples expressing the FLAG-tagged SDM proteins. For plasmid details, see Supplementary Table S3.

AbiEii is a DUF1814 protein and this group is further subdivided into COG2253 and COG4849 families. DUF1814 proteins contain four conserved motifs (Figure 8A). Motifs I and II at the N-terminus comprise the catalytic motif of DNA polymerase β (polβ) proteins with the hG[G/S]x_9__–__13_DhD motif (where h is hydrophobic and x is any amino acid). Alignment of AbiEii/DUF1814 representatives with DNA polβ proteins showed conservation of motifs I and II, but the spacing was longer than the consensus (*x* = 15 cf. 9–13) (Figure 8B). In addition, AbiEii/DUF1814 proteins contain an arginine within motif II, which is absent in other DNA polβ proteins (Figure 8B). The glycine and aspartates in motifs I and II are proposed to co-ordinate a metal-ion, most likely Mg^2+^, and assist nucleotide binding and transfer in a similar manner to polynucleotide polymerases (57,58). Therefore, DUF1814 proteins are hypothesized to bind a divalent metal (e.g. Mg^2+^) and catalyse a nucleotide transfer reaction similar to DNA polβ NTases. DUF1814 also contains a family-specific motif III, located at the C-terminus with a lysine surrounded by hydrophobic residues (Figure 8A and C). In the COG4849 family, motif III (KLxAaxxR, where *a* is any aromatic residue and *x* is any other amino acid) (53) is similar to the RxxRxxR motif of tRNA NTases, which led to the proposal that this motif mediates base stacking interactions for incoming nucleotides (53,59). In addition, the aromatic and basic amino acids might facilitate binding of the sugar or base of the nucleotide, similar to polymerases (57). We identified a motif specific to the AbiEii/DUF1814 proteins that has an acidic +DxxD pentad (+ denotes a positive charge (K or R) and x is any amino acid) (Figure 8A and C). This motif, termed motif IV, has no known function. We propose motifs III and IV are also involved in NTase activity since their location, based on homology models, is within the vicinity of the proposed catalytic centre (Supplementary Figure S10A and B).

### Mutations of conserved residues in AbiEii eliminate toxicity

To determine the role of motifs I-IV, and hence NTase toxic activity, site-directed mutagenesis (SDM) of AbiEii was performed. Variants were cloned with or without a C-terminal FLAG tag. A G49A, G50A double mutant and a single G49A motif I mutant were non-toxic, whereas a G50A variant was still toxic (Figure 8D). Indeed, G49 is conserved in DNA polβ proteins, whereas G50 is substituted with a serine or alanine in some homologues (Figure 8B). Mutations within motif II (R63A, D67A, D69A and a D67A/D69A double mutant), proposed to eliminate the metal-ion co-ordination that is essential for NTase catalysis, abrogated AbiEii function (Figure 8D). Conservative replacement of motif II aspartates with glutamates (D67E and D69E) abolished toxicity, suggesting the size difference might disfavour metal-ion co-ordination at the catalytic centre, resulting in non-toxic variants (Figure 8D). K173A or R180A substitutions in motif III also eliminated AbiEii toxicity, potentially by disrupting binding of nucleotide bases (Figure 8A and D). A D192A mutation within motif IV, which we propose is involved in nucleotide binding, also abolished toxicity (Figure 8D). All non-functional proteins were expressed, whereas the WT AbiEii and partially functional G50A proteins were not detected due to their toxicity (Figure 8D). The tagged AbiEii variants had the same phenotypes as the untagged versions (Supplementary Figure S10C). Overall, mutagenesis of the catalytic motifs of the DNA polβ family (motifs I and II) and motifs III and IV (exclusive to the AbiEii/DUF1814 family) abolished toxicity. Therefore, these residues are critical for AbiEii function, presumably via participating in a toxic NTase activity.

### AbiEii specifically binds GTP

**Figure 9.**
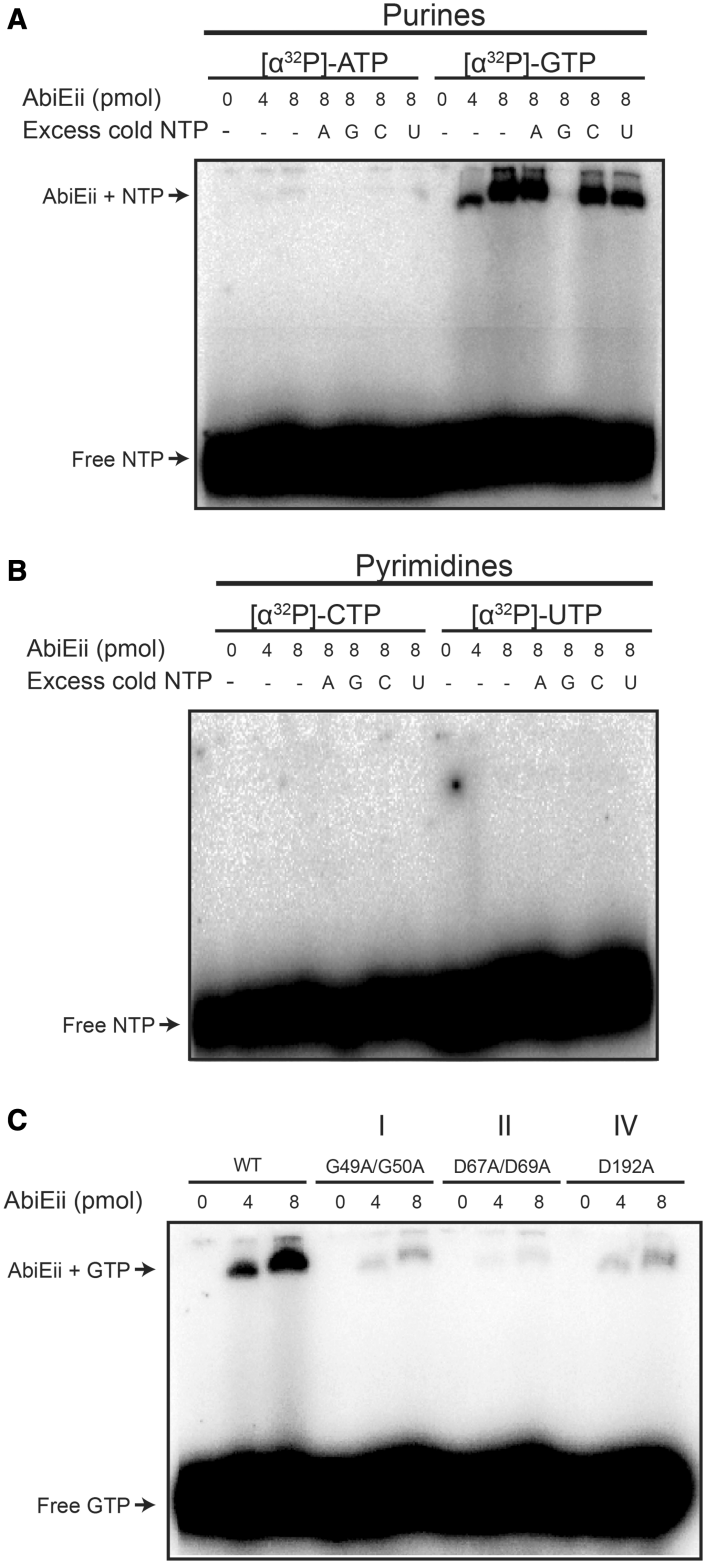
AbiEii specifically binds to GTP. (**A–B**) Increasing concentrations of AbiEii-His_6_ were incubated with 0.65 pmol of the indicated [α-^32^P]-radioactive nucleotides and analysed by native 6% TAE-PAGE. Nucleotide binding competition was conducted with the addition of excess (65 μmol) unlabelled nucleotide as indicated. (**C**) Purified AbiEii-His_6_ variants (motif I, G49/50A; motif II, D67A/D69A; and motif IV, D192A) were assayed for their ability to bind [α-^32^P]-GTP.

NTases transfer nucleoside monophosphate moiety (NMP) from NTP to a hydroxyl group on a target. To test if AbiEii was an NTase that bound nucleotides, AbiEii-His_6_ was incubated with [α-^32^P]-NTP and protein–nucleotide complexes were separated from free nucleotides on native polyacrylamide gels. Increasing AbiEii-His_6_ concentrations led to retardation in GTP migration, demonstrating GTP binding (Figure 9A). Limited binding to ATP was also observed (Figure 9A). GTP binding was specific, since competition with excess unlabelled GTP eliminated [α-^32^P]-ATP or [α-^32^P]-GTP binding, but other nucleotides had no effect (Figure 9A). AbiEii-His_6_ did not bind to either pyrimidines ([α-^32^P]-CTP or [α-^32^P]-UTP) (Figure 9B). Therefore, AbiEii binds purines, with a specificity and preference for GTP.

We hypothesized that mutations within the AbiEii catalytic motifs would affect nucleotide binding, and subsequent transfer to its substrate. Therefore, AbiEii-His_6_ variants with mutations in motif I (G49A/G50A), motif II (D67A/D69A) and motif IV (D192A) were tested for GTP binding. Alanine substitutions within motifs I and II (hG[G/S]x_15_DhD; as underlined) greatly reduced GTP binding (Figure 9C). The D192A substitution also severely alleviated GTP binding (Figure 9C), providing the first evidence that motif IV has a role in NTP binding. Therefore, the ability of AbiEii to bind GTP correlates with the ability to cause growth inhibition and is consistent with an NTase activity that elicits cytotoxicity.

## DISCUSSION

There is growing awareness of the importance of phage-host interactions and the role of resistance systems in this dynamic relationship. In addition, the widespread distribution and importance of TA loci in biological processes, including phage resistance, is becoming increasingly apparent. The discovery of ToxIN provided the first direct evidence of a link between Abi and TA systems (9). We sought to increase our mechanistic understanding of Abis and use them in a functional screen to identify new TA systems. Using an experimental model with inducible vectors in *E. coli*, we tested multiple lactococcal Abis and homologues for toxicity. This led to the identification of a widespread family of AbiE systems that function as TAs. AbiEii was toxic by acting as a GTP-binding NTase and was neutralized by expression of AbiEi, which is a novel DNA-binding protein that autoregulates the *abiE* operon. The identification of AbiE, in addition to ToxIN/AbiQ, demonstrates that a subset of Abis function as TAs.

AbiE constitutes a new Type IV TA system composed of a DUF1814 (COG2253) family bacteriostatic toxin, which was neutralized by the COG5340-AbiEi family. These systems are widespread in bacterial and archaeal genomes and on mobile genetic elements (MGEs). Based on the two Type IV systems discovered, this type is defined as possessing non-interacting toxin and antitoxins, in contrast to the Type II TA complexes. Indeed, no AbiEi–AbiEii interaction was observed in Co-IP and co-affinity protein purification experiments. The defining Type IV TA is CbeA-CbtA from *E. coli* that controls polymerization of cytoskeletal proteins MreB and FtsZ (21,60). A second system, CptB-CptA (YgfY/SdhE-YgfX) was reported as a Type IV TA (61); however, we were previously unable to detect a TA phenotype, despite observing protein–protein interactions for this system (62). Other studies of Type II TAs often do not include analysis of protein interactions and therefore, some might be Type IV loci.

The *abiE* genes are operonic and autoregulated, providing the first evidence that Type IV TAs can share these features in common with Type II systems. AbiEi (COG5340) proteins possess a novel antitoxin-fold with an N-terminal wHTH and an uncharacterized CTD. The wHTH resembles other DNA-binding domains in proteins such as BirA (biotin repressor) (63) and ArgR (arginine repressor) (64). AbiEi binds separately to two 23 bp IR elements spaced by 3 bp (i.e. 23-3-23) and represses *abiE* transcription. These binding sites cover the –35, –10, +1 and the downstream region melted during transcription initiation. Typically, DNA-binding antitoxins are dimeric and have protein folds including helix-turn-helix (MqsA), ribbon-helix-helix (RelE), loop-hinged-helix (MazE) and Phd/YefM-like (65). In contrast, AbiEi is monomeric, based on the structure of the related uncharacterized COG5340 protein Rv2827c (55). The surface of Rv2827c, and AbiEi (Supplementary Figure S11), containing recognition helix H3, is positively charged and extends along the uncharacterized CTD. Modelling suggested that upon rotation of the CTD, the full-length of Rv2827c might bind DNA (55). Based on the combined data, we predict that one AbiEi monomer binds independently to each 23 bp half site, and propose that the wHTH binds in the highly conserved (∼11 bp) short IR (Supplementary Figure S11). The CTD is proposed to interact with the remaining portion of the 23 bp ‘AbiEi-box’. Consistent with this model, deletion of various lengths of the CTD all impaired *abiE* promoter repression, suggesting that the full-length protein is required for DNA binding and repression. In contrast, the CTD alone provided complete antitoxicity, suggesting this domain is bi-functional—involved in both regulation and sufficient for protection. In agreement with the AbiEii toxin not regulating *abiE*, the autoregulation by AbiEi did not follow the conditional cooperativity model of Type II antitoxins (66). Our data provides the first evidence that Type IV antitoxins can autoregulate and the first detailed DNA binding and regulatory analysis of a COG5340 protein.

AbiEii induces bacteriostasis and is a predicted DNA polβ superfamily protein, which includes characterized and predicted NTases. Until this study, no detailed mutational or biochemical analysis had been performed on a DUF1814 protein. AbiEii/DUF1814 proteins contain conserved motifs (I–III) and we identified a new motif (IV). Individual mutation of each motif abolished toxicity. Motifs I and II are associated with the DNA polβ catalytic centre that involves metal-ion co-ordination for the NTase activity. AbiEii was shown to specifically bind GTP, the first step in a NTase activity. Motifs I, II and IV, were essential for optimal GTP binding and toxicity, demonstrating a nucleotide binding role for motif IV. All data suggest that AbiEii functions as a GTP-specific NTase that transfers this nucleotide to a target, resulting in growth inhibition. The DNA polβ superfamily has diverse functions in archaea, bacteria and eukaryotes (54). While DNA polβ members are proposed to have originated from the same precursor minimal NTase (MNT), accessory domains have partnered with the precursor MNT through evolution, diversifying the range of NTase substrates and targets (28). Therefore, predicting the DUF1814 target is challenging and could include RNA, DNA, protein, another nucleotide or a small molecule. The ability of the *L. lactis* and *S. agalactiae* AbiE systems to function in *E. coli* indicates that the target is highly conserved and its identification is part of our ongoing studies.

A number of possible models of NTase catalysis may result in toxicity, whereby AbiEii transfers one or more GMP(s) to its molecular target(s). We think it is unlikely that DNA is the target because of the reversible bacteriostatic mechanism of AbiEii. We propose that the addition of GMP would inactivate the function, and/or trigger the degradation of the target. Although unrelated to DNA polβ proteins, a recently characterized Type II TA-termed VbhAT from *Bartonella schoenbuchensis* was shown to elicit toxicity in *E. coli* through adenylylation of an unknown protein (67). Alternatively, AbiEii might function like polyA polymerase (PAP) or tRNA NTase (CCA adding) enzymes, to catalyse the transfer of GMP(s) to RNA(s). For example, PAP I from *E. coli* polyadenylates tRNAs when upregulated, leading to decreased aminoacylated tRNAs and subsequent protein synthesis, resulting in cell death (68). In addition, AbiEii may catalyse guanylylation of another nucleotide to produce an ‘alarmone’ that triggers growth arrest. For example, eukaryotic 2′-5′-oligoadenylyate synthetase (OAS) catalyses the synthesis of 2′-5′ oligoadenylate (2-5 A). The 2-5 A alarmone confers viral protection by binding and activation of the endoribonuclease RNaseL, which degrades RNA and halts viral propagation. Interestingly, OAS and AbiEii represent highly divergent members of DNA polβ proteins that provide viral evasion via NTase activity. The AbiEi antitoxin inhibits AbiEii-mediated toxicity and the CTD is sufficient for this activity. Given that AbiEi and AbiEii are non-interacting, the antitoxin CTD might catalyse the removal of the GMP(s) from the target(s). These models require further investigation.

A question of interest in the TA field is what are the biological functions of TA systems (16,18). AbiE of *L. lactis* provides resistance against the 936 phage family by preventing DNA packaging, but infected cells do not survive (36,47,48). Since AbiEii is toxic in the absence of phage infection, the prevention of phage DNA packaging might be an indirect effect of the phage triggering cellular, and phage, arrest via activation of the AbiEii toxin. A recent study has confirmed that a TA system containing a DUF1814 protein, termed *sanaTA* from *Shewanella* sp., provides phage T7 resistance to *E. coli* (69). Resistance required mutation of the T7 gene gp4.5, which encodes a protein that interacts with, and might inhibit, Lon protease. Presumably, gp4.5 blocks Lon-mediated antitoxin degradation, thus prevents TA activation (69). In *L. lactis*, AbiE is encoded by a conjugative mega-plasmid, pNP40 (36), and in *S. agalactiae abiE* is located on an ICE, ICE*Sa2603rplL* (49). We identified AbiE systems situated in MGEs, many of which encode other genes for antibiotic or heavy metal resistance (Supplementary Table S4). Therefore, in addition to phage resistance, AbiE loci might have other roles, such as MGE maintenance. In agreement, recent analysis of defense islands in bacteria and archaea revealed an abundance of COG5340–DUF1814 gene pairs and it was speculated that these might encode TA systems (70,71). Indeed, we showed AbiE stabilized an unstable plasmid (Figure 4A), and therefore might be involved in stabilization of ICE*Sa2603rplL*. ToxIN is another example of an Abi-TA system that has dual-roles in phage resistance and for plasmid stabilization (9,14). Furthermore, the MosAT TA system in *Vibrio cholerae* is situated on the SXT ICE, and promotes island maintenance (72). SXT provides *V. cholerae* isolates with multi-drug resistance. Our analyses of MosAT indicate these proteins constitute a COG2253–COG5340 TA system similar to AbiE (Figure 3A). Thus, AbiE represents a widely disseminated TA-Abi system in bacteria that can enable maintenance of plasmids and other MGEs and provide phage resistance. It is unknown whether all systems provide dual stabilization/phage resistance functions.

In conclusion, we have identified and characterized a new widespread Type IV TA family. This system is autoregulated by the antitoxin via a mechanism distinct from known Type II antitoxins. Toxicity is bacteriostatic, mediated by the GTP-specific NTase domain of the toxin. In addition, the antitoxin CTD is sufficient for inactivation of toxicity. The future challenge is to identify the target(s) of these NTase(s). Along with the ToxIN/AbiQ systems, we have provided additional evidence that the molecular action of some Abis can be via a TA mechanism. Equally, particular TAs provide phage resistance, emphasizing the functional similarity between these diverse and abundant loci. Given the abundance of bacteriophages and these Abi/TA systems, understanding their molecular mechanism(s) and evolutionary drivers warrants further investigation.

## SUPPLEMENTARY DATA

Supplementary Data are available at NAR Online.

## FUNDING

The Marsden Fund and a Rutherford Discovery Fellowship (to P.C.F.) both from the Royal Society of New Zealand; a University of Otago Postgraduate Scholarship, an Otago Postgraduate Publishing Bursary and a Sandy Smith Memorial Scholarship (to R.L.D.). Funding for open access: The Marsden Fund and a Rutherford Discovery Fellowship (to P.C.F.) both from the Royal Society of New Zealand.

*Conflict of interest statement*. None declared.

## References

[1] Weinbauer MG, Rassoulzadegan F (2004). Are viruses driving microbial diversification and diversity?. Environ. Microbiol..

[2] Wommack KE, Colwell RR (2000). Virioplankton: viruses in aquatic ecosystems. Microbiol. Mol. Biol. Rev..

[3] Chibani-Chennoufi S, Bruttin A, Dillmann ML, Brussow H (2004). Phage-host interaction: an ecological perspective. J. Bacteriol..

[4] Chopin MC, Chopin A, Bidnenko E (2005). Phage abortive infection in lactococci: variations on a theme. Curr. Opin. Microbiol..

[5] Labrie SJ, Samson JE, Moineau S (2010). Bacteriophage resistance mechanisms. Nat. Rev. Microbiol..

[6] Richter C, Chang JT, Fineran PC (2012). Function and regulation of clustered regularly interspaced short palindromic repeats (CRISPR) / CRISPR associated (Cas) systems. Viruses.

[7] Shub DA (1994). Bacterial viruses. Bacterial altruism?. Curr. Biol..

[8] Yarmolinsky MB (1995). Programmed cell death in bacterial populations. Science.

[9] Fineran PC, Blower TR, Foulds IJ, Humphreys DP, Lilley KS, Salmond GP (2009). The phage abortive infection system, ToxIN, functions as a protein-RNA toxin-antitoxin pair. Proc. Natl Acad. Sci. USA.

[10] Blower TR, Fineran PC, Johnson MJ, Toth IK, Humphreys DP, Salmond GP (2009). Mutagenesis and functional characterization of the RNA and protein components of the *toxIN* abortive infection and toxin-antitoxin locus of *Erwinia*. J. Bacteriol..

[11] Blower TR, Short FL, Rao F, Mizuguchi K, Pei XY, Fineran PC, Luisi BF, Salmond GP (2012). Identification and classification of bacterial Type III toxin-antitoxin systems encoded in chromosomal and plasmid genomes. Nucleic Acids Res..

[12] Emond E, Dion E, Walker SA, Vedamuthu ER, Kondo JK, Moineau S (1998). AbiQ, an abortive infection mechanism from *Lactococcus lactis*. Appl. Environ. Microbiol..

[13] Blower TR, Pei XY, Short FL, Fineran PC, Humphreys DP, Luisi BF, Salmond GP (2011). A processed noncoding RNA regulates an altruistic bacterial antiviral system. Nat. Struct. Mol. Biol..

[14] Short FL, Pei XY, Blower TR, Ong SL, Fineran PC, Luisi BF, Salmond GP (2013). Selectivity and self-assembly in the control of a bacterial toxin by an antitoxic noncoding RNA pseudoknot. Proc. Natl Acad. Sci. USA.

[15] Samson JE, Spinelli S, Cambillau C, Moineau S (2013). Structure and activity of AbiQ, a lactococcal endoribonuclease belonging to the type III toxin-antitoxin system. Mol. Microbiol..

[16] Gerdes K, Maisonneuve E (2012). Bacterial persistence and toxin-antitoxin loci. Annu. Rev. Microbiol..

[17] Cook GM, Robson JR, Frampton RA, McKenzie J, Przybilski R, Fineran PC, Arcus VL (2013). Ribonucleases in bacterial toxin-antitoxin systems. Biochim. et Biophys. Acta.

[18] Van Melderen L (2010). Toxin-antitoxin systems: why so many, what for?. Curr. Opin. Microbiol..

[19] Fozo EM, Hemm MR, Storz G (2008). Small toxic proteins and the antisense RNAs that repress them. Microbiol. Mol. Biol. Rev..

[20] Leplae R, Geeraerts D, Hallez R, Guglielmini J, Dreze P, Van Melderen L (2011). Diversity of bacterial type II toxin-antitoxin systems: a comprehensive search and functional analysis of novel families. Nucleic Acids Res..

[21] Masuda H, Tan Q, Awano N, Wu KP, Inouye M (2012). YeeU enhances the bundling of cytoskeletal polymers of MreB and FtsZ, antagonizing the CbtA (YeeV) toxicity in *Escherichia coli*. Mol. Microbiol..

[22] Wang X, Lord DM, Cheng HY, Osbourne DO, Hong SH, Sanchez-Torres V, Quiroga C, Zheng K, Herrmann T, Peti W (2012). A new type V toxin-antitoxin system where mRNA for toxin GhoT is cleaved by antitoxin GhoS. Nat. Chem. Biol..

[23] Blower TR, Evans TJ, Przybilski R, Fineran PC, Salmond GP (2012). Viral evasion of a bacterial suicide system by RNA-based molecular mimicry enables infectious altruism. PLoS Genet..

[24] Hazan R, Engelberg-Kulka H (2004). *Escherichia coli mazEF*-mediated cell death as a defense mechanism that inhibits the spread of phage P1. Mol. Genet. Genomics.

[25] Pecota DC, Wood TK (1996). Exclusion of T4 phage by the *hok/sok* killer locus from plasmid R1. J. Bacteriol..

[26] Otsuka Y, Yonesaki T (2012). Dmd of bacteriophage T4 functions as an antitoxin against *Escherichia coli* LsoA and RnlA toxins. Mol. Microbiol..

[27] Fozo EM, Makarova KS, Shabalina SA, Yutin N, Koonin EV, Storz G (2010). Abundance of type I toxin-antitoxin systems in bacteria: searches for new candidates and discovery of novel families. Nucleic Acids Res..

[28] Makarova KS, Wolf YI, Koonin EV (2009). Comprehensive comparative-genomic analysis of type 2 toxin-antitoxin systems and related mobile stress response systems in prokaryotes. Biol. Direct.

[29] Guglielmini J, Szpirer C, Milinkovitch MC (2008). Automated discovery and phylogenetic analysis of new toxin-antitoxin systems. BMC Microbiol..

[30] Sambrook J, Fritsch E, Maniatis T (1989). Molecular Cloning: A Laboratory Manual.

[31] Lodge J, Fear J, Busby S, Gunasekaran P, Kamini NR (1992). Broad host range plasmids carrying the *Escherichia coli* lactose and galactose operons. FEMS Microbiol. Lett..

[32] Przybilski R, Richter C, Gristwood T, Clulow JS, Vercoe RB, Fineran PC (2011). Csy4 is responsible for CRISPR RNA processing in *Pectobacterium atrosepticum*. RNA Biol..

[33] Miller JH (1972). Experiments in Molecular Genetics.

[34] Heckman KL, Pease LR (2007). Gene splicing and mutagenesis by PCR-driven overlap extension. Nat. Protoc..

[35] Deng YM, Liu CQ, Dunn NW (1999). Genetic organization and functional analysis of a novel phage abortive infection system, AbiL, from *Lactococcus lactis*. J. Biotechnol..

[36] Garvey P, Fitzgerald GF, Hill C (1995). Cloning and DNA sequence analysis of two abortive infection phage resistance determinants from the lactococcal plasmid pNP40. Appl. Environ. Microbiol..

[37] O'Connor L, Coffey A, Daly C, Fitzgerald GF (1996). AbiG, a genotypically novel abortive infection mechanism encoded by plasmid pCI750 of *Lactococcus lactis* subsp. *cremoris* UC653. Appl. Environ. Microbiol..

[38] O'Connor L, Tangney M, Fitzgerald GF (1999). Expression, regulation, and mode of action of the AbiG abortive infection system of *Lactococcus lactis* subsp. *cremoris* UC653. Appl. Environ. Microbiol..

[39] Bouchard JD, Dion E, Bissonnette F, Moineau S (2002). Characterization of the two-component abortive phage infection mechanism AbiT from *Lactococcus lactis*. J. Bacteriol..

[40] Dai G, Su P, Allison GE, Geller BL, Zhu P, Kim WS, Dunn NW (2001). Molecular characterization of a new abortive infection system (AbiU) from *Lactococcus lactis* LL51-1. Appl. Environ. Microbiol..

[41] Anba J, Bidnenko E, Hillier A, Ehrlich D, Chopin MC (1995). Characterization of the lactococcal *abiD1* gene coding for phage abortive infection. J. Bacteriol..

[42] Bidnenko E, Chopin A, Ehrlich SD, Chopin MC (2009). Activation of mRNA translation by phage protein and low temperature: the case of *Lactococcus lactis* abortive infection system AbiD1. BMC Mol. Biol..

[43] Emond E, Holler BJ, Boucher I, Vandenbergh PA, Vedamuthu ER, Kondo JK, Moineau S (1997). Phenotypic and genetic characterization of the bacteriophage abortive infection mechanism AbiK from *Lactococcus lactis*. Appl. Environ. Microbiol..

[44] Prevots F, Tolou S, Delpech B, Kaghad M, Daloyau M (1998). Nucleotide sequence and analysis of the new chromosomal abortive infection gene *abiN* of *Lactococcus lactis* subsp. *cremoris* S114. FEMS Microbiol. Lett..

[45] Prevots F, Ritzenthaler P (1998). Complete sequence of the new lactococcal abortive phage resistance gene *abiO*. J. Dairy Sci..

[46] Yang JM, Deurraza PJ, Matvienko N, O'Sullivan DJ (2006). Involvement of the LlaKR2I methylase in expression of the AbiR bacteriophage defense system in *Lactococcus lactis* subsp. *lactis* biovar *diacetylactis* KR2. J. Bacteriol..

[47] Garvey P, Rince A, Hill C, Fitzgerald GF (1997). Identification of a *recA* homolog (recALP) on the conjugative lactococcal phage resistance plasmid pNP40: evidence of a role for chromosomally encoded *recAL* in abortive infection. Appl. Environ. Microbiol..

[48] Tangney M, Fitzgerald GF (2002). AbiA, a lactococcal abortive infection mechanism functioning in *Streptococcus thermophilus*. Appl. Environ. Microbiol..

[49] Haenni M, Saras E, Bertin S, Leblond P, Madec JY, Payot S (2010). Diversity and mobility of integrative and conjugative elements in bovine isolates of *Streptococcus agalactiae, S. dysgalactiae subsp. dysgalactiae,* and *S.uberis. Appl. Environ*. Microbiol..

[50] Palmieri C, Magi G, Creti R, Baldassarri L, Imperi M, Gherardi G, Facinelli B (2013). Interspecies mobilization of an *erm*(T)-carrying plasmid of *Streptococcus dysgalactiae* subsp. *equisimilis* by a coresident ICE of the ICE*Sa2603* family. J. Antimicrob. Chemother..

[51] Brochet M, Couve E, Glaser P, Guedon G, Payot S (2008). Integrative conjugative elements and related elements are major contributors to the genome diversity of *Streptococcus agalactiae*. J. Bacteriol..

[52] Franceschini A, Szklarczyk D, Frankild S, Kuhn M, Simonovic M, Roth A, Lin J, Minguez P, Bork P, von Mering C (2013). STRING v9.1: protein-protein interaction networks, with increased coverage and integration. Nucleic Acids Res..

[53] Kim BH, Sadreyev R, Grishin NV (2005). COG4849 is a novel family of nucleotidyltransferases. J. Mol. Recognit..

[54] Kuchta K, Knizewski L, Wyrwicz LS, Rychlewski L, Ginalski K (2009). Comprehensive classification of nucleotidyltransferase fold proteins: identification of novel families and their representatives in human. Nucleic Acids Res..

[55] Janowski R, Panjikar S, Eddine AN, Kaufmann SH, Weiss MS (2009). Structural analysis reveals DNA binding properties of Rv2827c, a hypothetical protein from *Mycobacterium tuberculosis*. J. Struct. Funct. Genomics.

[56] Aravind L, Anantharaman V, Balaji S, Babu MM, Iyer LM (2005). The many faces of the helix-turn-helix domain: transcription regulation and beyond. FEMS Microbiol. Rev..

[57] Martin G, Keller W (1996). Mutational analysis of mammalian poly(A) polymerase identifies a region for primer binding and catalytic domain, homologous to the family X polymerases, and to other nucleotidyltransferases. EMBO J..

[58] Steitz TA (1997). DNA and RNA polymerases: structural diversity and common mechanisms. Harvey Lect..

[59] Li F, Xiong Y, Wang J, Cho HD, Tomita K, Weiner AM, Steitz TA (2002). Crystal structures of the *Bacillus stearothermophilus* CCA-adding enzyme and its complexes with ATP or CTP. Cell.

[60] Tan Q, Awano N, Inouye M (2011). YeeV is an *Escherichia coli* toxin that inhibits cell division by targeting the cytoskeleton proteins, FtsZ and MreB. Mol. Microbiol..

[61] Masuda H, Tan Q, Awano N, Yamaguchi Y, Inouye M (2012). A novel membrane-bound toxin for cell division, CptA (YgfX), inhibits polymerization of cytoskeleton proteins, FtsZ and MreB, in *Escherichia coli*. FEMS Microbiol. Lett..

[62] McNeil MB, Iglesias Cans M, Clulow JS, Fineran P (2013). YgfX (CptA) is a multimeric membrane protein that interacts with the succinate dehydrogenase assembly factor SdhE (YgfY). Microbiology.

[63] Streaker ED, Beckett D (1998). A map of the biotin repressor-biotin operator interface: binding of a winged helix-turn-helix protein dimer to a forty base-pair site. J. Mol. Biol..

[64] Sunnerhagen M, Nilges M, Otting G, Carey J (1997). Solution structure of the DNA-binding domain and model for the complex of multifunctional hexameric arginine repressor with DNA. Nat. Struct. Biol..

[65] Hayes F, Van Melderen L (2011). Toxins-antitoxins: diversity, evolution and function. Crit. Rev. Biochem. Mol..

[66] Overgaard M, Borch J, Jorgensen MG, Gerdes K (2008). Messenger RNA interferase RelE controls *relBE* transcription by conditional cooperativity. Mol. Microbiol..

[67] Engel P, Goepfert A, Stanger FV, Harms A, Schmidt A, Schirmer T, Dehio C (2012). Adenylylation control by intra- or intermolecular active-site obstruction in Fic proteins. Nature.

[68] Mohanty BK, Kushner SR (2013). Deregulation of poly(A) polymerase I in *Escherichia coli* inhibits protein synthesis and leads to cell death. Nucleic Acids Res..

[69] Sberro H, Leavitt A, Kiro R, Koh E, Peleg Y, Qimron U, Sorek R (2013). Discovery of functional toxin/antitoxin systems in bacteria by shotgun cloning. Mol. Cell.

[70] Makarova KS, Wolf YI, Koonin EV (2013). Comparative genomics of defense systems in archaea and bacteria. Nucleic Acids Res..

[71] Makarova KS, Wolf YI, Snir S, Koonin EV (2011). Defense islands in bacterial and archaeal genomes and prediction of novel defense systems. J. Bacteriol..

[72] Wozniak RA, Waldor MK (2009). A toxin-antitoxin system promotes the maintenance of an integrative conjugative element. PLoS Genet..

